# The methyltransferase activity of Dot1L is essential for *Xenopus tropicalis* tadpole development and survival

**DOI:** 10.1186/s13578-026-01537-8

**Published:** 2026-03-03

**Authors:** Emeric M. Louis, Nga Luu, Laurent M. Sachs, Yun-Bo Shi

**Affiliations:** 1 Section on Molecular Morphogenesis, Eunice Kennedy Shriver National Institute of Child Health and Human Development, National Institutes of Health, Bethesda, MD USA; 2 UMR 7221 Physiologie Moléculaire et Adaptation, CNRS, Muséum National d’Histoire Naturelle, Alliance Sorbonne Universités, CP32, 57 rue Cuvier, 75005 Paris, France

**Keywords:** Histone methyltransferase, H3K79 methylation, Epigenetics, Thyroid hormone, Amphibians

## Abstract

Epigenetic modifications of histones play key roles in transcriptional regulation by diverse transcription factors. One such modification is the methylation of histone H3 at lysine 79 (H3K79). The methyltransferase DOT1L is the only known, highly conserved enzyme capable of methylating H3K79 in a wide range of species. We have been studying the role of DOT1L during *Xenopus tropicalis* development. We have generated a *Dot1L* mutant line with a deletion of 6 amino acids and two amino acid substitutions in the catalytic domain, resulting in a complete loss of H3K79 methylation in the developing animals. Importantly, we have demonstrated that the absence of H3K79 methylation during *X. tropicalis* development leads to lethality at or before stage 48 (about 30 days post-fertilization), as well as delayed growth and developmental progression in tadpoles. These findings highlight a crucial role for H3K79 methylation during premetamorphic development of *X. tropicalis*.

**Supplementary Information:**

The online version contains supplementary material available at 10.1186/s13578-026-01537-8.

**Dear Editor,**

Epigenetic modifications play a key role in all metazoan development. Histones are subjected to numerous post-translational modifications, including acetylation, methylation, phosphorylation, and ubiquitylation [1]. Among them, methylation of histone H3 at lysine 79 (H3K79) is associated with high levels of gene expression and methylated H3K79 (H3K79me) is considered an activation histone mark [2]. This modification occurs in the globular domain of histone H3 and is known to be catalyzed only by the Disruptor of Telomeric Silencing 1-like (Dot1L). Originally discovered as Dot1 in *Saccharomyces cerevisiae* [3], Dot1L dysfunction has recently been shown to cause a congenital neuronal disorder in human [4]. Moreover, Dot1L was identified as a direct target of thyroid hormone (TH) receptor (TR) during TH-dependent development metamorphosis in *Xenopus tropicalis* [5], which mirrors post-embryonic development in mammals, corresponding to the perinatal period in human. Notably, H3K79 methylation increases at TR target genes following TH treatment or during natural metamorphosis, when endogenous TH levels are elevated [6]. Furthermore, transgenic overexpression and gene knockdown studies have shown that Dot1L can function as a TR co-activator during *Xenopus* development [7]. Interestingly, Dot1L knockdown with TALEN genome editing also revealed that all F0 generation tadpoles obtained from injecting a TALEN targeting Dot1L catalytic domain into fertilized *Xenopus tropicalis* eggs died at tadpole stages before reaching the onset of metamorphosis (stage 54) with a loss of H3K79 methylation proportional to the fractions of Dot1L gene mutated in the F0 tadpoles, suggesting that Dot1L is the only enzyme capable of methylating H3K79 in developing tadpoles and that Dot1L is required for tadpole survival before metamorphosis [7].

To better understand the role of Dot1L, particular its methyltransferase activity, during development, we obtained a stable heterozygote transgenic line by using the F0 adult frogs generated from TALEN genome editing [8]. This line, Dot1L^Δ18^, had a specific mutation in the Dot1L gene that included 18 bp in-frame deletion and two additional adjacent nucleotide substitutions in the region encoding the methyltransferase domain, leading to a substitution of two amino acids and a deletion of six amino acids (Fig. 1A). Not surprisingly, the in-frame deletion and substitution did not affect Dot1L expression in tadpoles (Fig. 1B). On the other hand, the mutation delayed tadpole growth and development in homozygous Dot1L^Δ18^ tadpoles, compared to wild type (WT) or heterozygous (HE) animals (Fig. 1C.

**Fig. 1:**
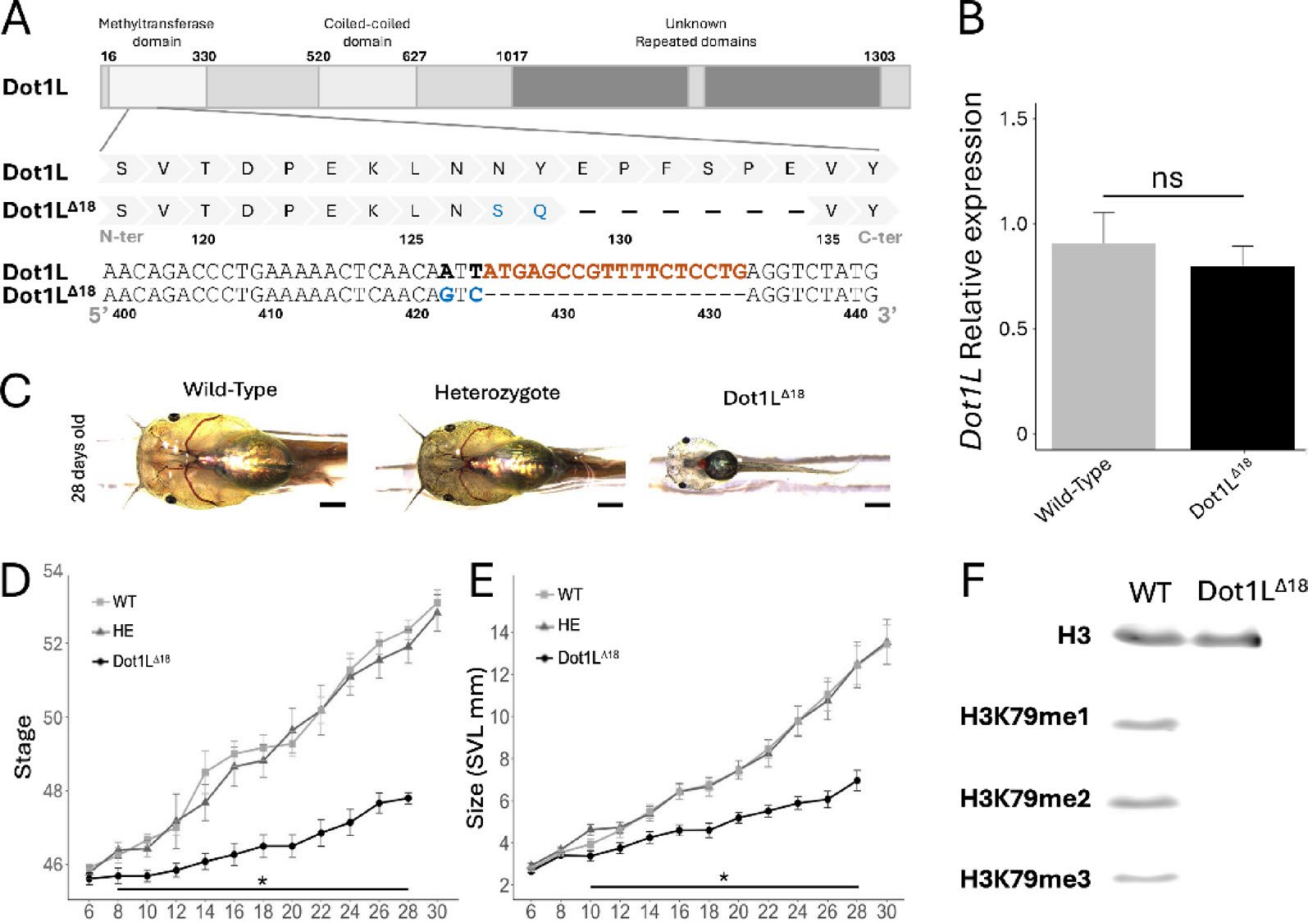
Dot1L^∆18^ mutants show a complete loss of H3K79 methylation. **A** Schematic representation of the Dot1L^∆18^ mutation located in the methyltransferase domain of Dot1L. **B**The expression of Dot1L mRNA is not affected by the mutation. Relative expression of *Dot1L* in wild type (WT) and Dot1L^∆18^ tadpoles at stage 48 was measured by qRT-PCR, normalized to that of eIFα. Note that no significant difference in *Dot1L* transcript levels was observed between the two genotypes. **C** Phenotypic observation at 28 days post-fertilization in WT, heterozygous (HE), and homozygous (Dot1L^∆18^) Dot1L^∆18^ animals. Twenty animals per genotype were housed individually after genotyping and photographed every 2 days, from day 6 to day 30 to measure the developmental stage (**D**) or *snout-to-Vent (SVL)* size (**E**). Bars represent standard derivations, and stars indicate a significant difference between homozygous Dot1L^∆18^ (Dot1L^∆18^) and the other two genotypes based on pairwise t-test (p.value < 0.05). **F** Western-blot analysis comparing H3 and methylated H3 in WT and Dot1L^∆18^ tadpoles at stage 48 by using antibodies against total H3, H3K79me1, H3K79me2, and H3K79me3. Note that no H3K79 methylation was detectable in Dot1L^∆18^ mutants

To further investigate the developmental effects of the mutation, we monitored 13 tadpoles per genotype (WT, HE, and Dot1L^Δ18^) from post-fertilization day 6 to day 30 (Fig. 1D,E). They were photographed every two days to assess their size and developmental stage according to the Nieuwkoop and Faber table [9]. At day 6, no significant differences were observed among the groups, suggesting that the mutation did not affect early development. However, Dot1L^Δ18^ tadpoles were significantly smaller than the other two groups by day 8 and had a developmental delay, reaching lower developmental stages by day 10 compared to the wild type or heterozygous siblings. This delay progressively worsened and all homozygous Dot1L^Δ18^ tadpoles died around stage 48 (~ 30 days post-fertilization).

The mutation in the catalytic domain is expected to affect the methyltransferase activity of Dot1L. To test this, we analyzed by western blot the levels of various H3K79 methylation in tadpoles at stage 48 (Fig. 1F). The results showed a complete absence of mono-, di-, and tri-methylated H3K79 in Dot1L^Δ18^ mutants. Thus, the mutation abolished H3K79 methylation activity in Dot1L and that Dot1L is the only methyltransferase responsible for H3K79 methylation in *Xenopus tropicalis* tadpoles.

During the studies presented in Fig. 1D, E, we noticed that the homozygous Dot1L^Δ18^ animals began to die by day 16. Quantification of the survival of 13 animals for each genotype in the studies showed that the death of the homozygous Dot1L^Δ18^ animals continued until all died by day 30 (Fig. 2A). To confirm this lethal phenotype, we performed a different analysis by using another batch of sibling animals. We followed the genotype distribution of a large batch of animals generated from mating of heterozygous frogs by genotyping randomly selected 30 tadpoles every other day from the batch (Fig. 2B). We observed that the genotype distribution followed Mendelian ratios until day 12. Subsequently, the proportion of Dot1L^Δ18^ tadpoles decreased, eventually to 0% by day 30, confirming that H3K79me is essential for tadpole viability during post-embryonic development.

**Fig. 2:**
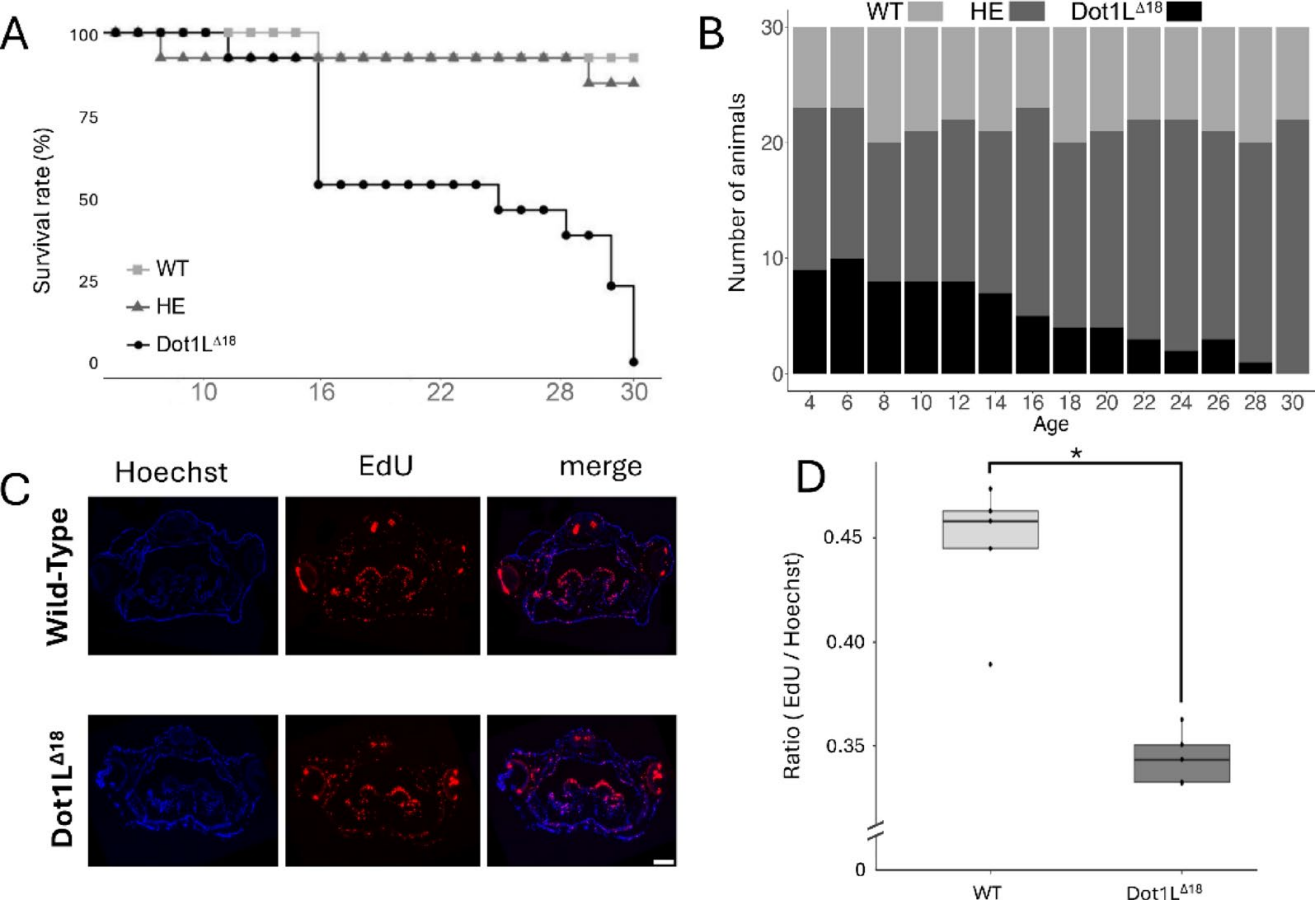
Loss of H3K79 methylation leads to mortality and reduced cell proliferation during *X. tropicalis* development. **A** Survival rate of wild type (WT), heterozygous (HE) and homozygous Dot1L^∆18^ tadpoles (13 animals per group). A progressive mortality was observed in Dot1L^∆18^ mutants from day 16 to day 30 post-fertilization, with no surviving individuals beyond this time point. **B** Genotype distribution of 30 tadpoles randomly sampled every 2 days from 108 sibling animals generated from mating of two HE adults, housed in a 9-L tank. Note that from day 4 to day 14, the genotype ratio followed Mendelian expectations; however, from day 16 onward, Dot1L^∆18^ tadpoles were gradually reduced to 0% by day 30, consisting with independent findings in (**A**). **C** Representative images of cell proliferation in the head region (median eye cross-section) of WT and Dot1L^∆18^ tadpoles, assessed with 16 h EdU incorporation. **D** Quantification of EdU-positive cells relative to DNA staining (EdU/Hoechst ratio) in six sections each from two WT and two Dot1L^∆18^ tadpoles. A t-test revealed a significant reduction in cell proliferation in Dot1L^∆18^ animals compared to WT animals (p.value = 0.001213)

The smaller size of the homozygous Dot1L^Δ18^ animals compared to age-matched wild type and heterozygous siblings suggests likely reduced cell proliferation in the Dot1L^Δ18^ animals. To test this, we used EdU to label proliferating cells in tadpoles at stage 48 and found significantly reduced cell proliferation in the head region of Dot1L^Δ18^ animals compared to wild type and heterozygous siblings (Fig. 2C, D). These findings suggest methylation by Dot1L plays a key role in *Xenopus* development by regulating cell proliferation, although it remains possible that that Dot1L may also affect cell death during development.

The morphological and cellular phenotypes that we reported here are consistent with our earlier observations with the F0 generation tadpoles generated by injecting TALEN targeting Dot1L catalytic domain into fertilized *Xenopus tropicalis* eggs [7]. Such F0 generation tadpoles had numerous mosaic mutations, most of which were out-of-frame deletions/insertions [7]. Here our mutant line has single mutation that leads to the production of mutant protein with a deletion of 6 amino acids and substitution of two additional adjacent amino acids within the catalytic domain (Fig. 1A). While we have not been able to measure the Dot1L protein level due to the lack of a proper antibody, it is expected that both wild type and mutant proteins are expressed at similar levels just like their mRNA levels. In addition, the methyltransferase activity of mutant Dot1L will be disrupted due to the deletion and substitutions of amino acids in the conserved methyltransferase domain critical for the structure and/or enzymatic activity of the enzyme. Indeed, our analyzes of methylated H3K79 in vivo confirmed the lack of H3K79 methylation activity during *Xenopus* development even though Dot1L expression was not affected. Our findings here and those from the F0 generation tadpoles reported earlier [7] thus complement each other to support the conclusion that the cellular and development effects of the mutation was due to the disruption of the methyltransferase activity of Dot1L, not due to any potential effects of mutant Dot1L. To our knowledge, this represents the first report of a major developmental role for the methyltransferase activity of Dot1L in any vertebrates. Future genome-wide and molecular studies, such mapping targets sites of Dot1L and/or H3K79 methylation via ChIP (chromatin-immunoprecipitation)-seq assay and assessing transcriptome changes change between WT and Dot1L^∆18^ tadpoles by using RNAseq or single cell/nucleus RNAseq should help to determine molecular pathways underlying the developmental functions of Dot1L.

## Supplementary Information

Below is the link to the electronic supplementary material.

Supplementary Material 1

## Data Availability

All data are available in the main text.
