# Supplementary material for: The methyltransferase activity of Dot1L is essential for Xenopus tropicalis tadpole development and survival
Source: Cell Biosci. 2026 Mar 3;16:26. doi: 10.1186/s13578-026-01537-8 (PMC12954907; doi:10.1186/s13578-026-01537-8)
Supplement: Supplementary file 1 — Supplementary Material 1 [file 13578_2026_1537_MOESM1_ESM.pdf]

SUPPLEMENTARY MATERIALS AND METHODS

Animal Rearing

All procedures followed the guidelines approved by the Animal Use and Care Committee (NICHD, NIH). Adult Xenopus tropicalis were purchased from the National Xenopus Resource. Staging was based on criteria for *Xenopus laevis*.

Genotyping

DNA was extracted from tail fragments using QuickExtract™ DNA Extraction Solution (Lucigen). Dot1L was amplified by PCR with primers (Forward: 5'-CCCCACTAATACAACAACCGGC-3', Reverse: 5'-AATGGGGGTGCCTAACCACT-3'). PCR products were analyzed on 3% agarose gels. Sequencing (Eurofins Genomics) was used for confirmation.

Survival Rates and Phenotyping

120 tadpoles obtained by mating heterozygous adults were genotyped at 6 days post fertilization (dpf), and 13 tadpoles per genotype were reared and observed in separate tanks. Tadpoles from an independent mating were used for imaging and growth measurements.

EdU Labeling for Cell Proliferation

Genotyped tadpoles were incubated in EdU solution (0.25 µg/mL) for 16 h, fixed in 4% paraformaldehyde (PFA), embedded, and sectioned. Staining was done with Click-iT® Alexa Fluor 594 Imaging kit (Invitrogen). Quantification was done with Photoshop.

RNA isolation

After sacrificed 3 wild-type or $Dot1L^{\Delta 18}$ animals (stage 48) in 500 µl of TRizol™ Reagent (Thermo Fisher Scientific), the mixture was spun in an Eppendorf centrifuge at 12000 rpm at 4°C for 15 min. The supernatant was extracted with 200 µl chloroform. The RNA was then purified with Direct-zol RNA MiniPrep kit (Zymo Research) and eluted in 30 µl nuclease-free water. RNA concentration was adjusted at 100 ng/µl and RNA quality was checked with Bioanalyzer RNA 6000 Nano kit (Agilent).

qRT-PCR

cDNA synthesis of was done with SuperScript™ III First-Strand Synthesis System (Invitrogen). The final products were analyzed in triplicates by qPCR with SYBR Green Master Mix Reagent (Thermo Fisher Scientific) and Dot1L primers (F: 5’-CATCCTATGGAAGTTGAAAACTCG-3’ and R: 5’-CAATCATCTGGGCAACCAAAT-3’) and normalized with the control gene eIF1α (F: 5’-CCCCTCTTGGTCGTTTTGCTGTCC-3’ and R: 5’-TTGCCTTTCTGTGCTTTCTGAGCAG-3’).

Protein Extraction and Western Blot

Tadpoles were lysed in RIPA buffer (Thermo Fisher Scientific) for protein isolation. The protein concentrations were measured with Bradford assay. The protein samples were separated by SDS-PAGE and transferred to membranes. Membranes were probed with

antibodies for H3K79me1 (ab2886, Abcam), H3K79me2 (ab3594, Abcam), H3K79me3 (ab2621, Abcam), or total H3 (PA5-16183, Thermo Fisher Scientific). Fluorescence imaging was done with a Li-Cor Odyssey Clx Imager.

Image and Statistical Analysis
Images of tissues sections were acquired with a Leica microscope. Quantitative analyses were done by using Adobe Photoshop. Statistical comparisons were performed by using the Kruskal-Wallis test or student test.
